# Case Report: Dental treatment under general anesthesia for a child with Ullrich congenital muscular dystrophy

**DOI:** 10.3389/fdmed.2026.1881573

**Published:** 2026-09-03

**Authors:** SokIok Cheang, WeiYun Chen, Shuang Wang, XinXin Wang, Kuo Wan, Lin Ma

**Affiliations:** 1 Department of Stomatology, Peking Union Medical College Hospital, Chinese Academy of Medical Science and Peking Union Medical College, Beijing, China; 2 Department of Anesthesiology, Peking Union Medical College Hospital, Chinese Academy of Medical Science and Peking Union Medical College, Beijing, China; 3 International Medical Services of Stomatology, Peking Union Medical College Hospital, Chinese Academy of Medical Science and Peking Union Medical College, Beijing, China

**Keywords:** case report, dental management, general anesthesia, perioperative period, Ullrich congenital muscular dystrophy

## Abstract

**Background:**

Ullrich congenital muscular dystrophy (UCMD) is a rare inherited myopathy caused by mutations in the COL6 gene and is frequently associated with multisystem complications affecting the respiratory and skeletal systems, which may increase airway and anesthetic risks during dental treatment.

**Case report:**

This report describes a 4-year-old girl with UCMD who presented with pain in the left lower molar. After a comprehensive multidisciplinary evaluation by the Department of Stomatology, Anesthesiology, and Pediatrics, the patient was classified as ASA III and underwent dental treatment under total intravenous anesthesia, with tracheal intubation performed under fiberoptic bronchoscopic guidance. The procedures included pulpotomy, tooth extraction, resin restoration, placement of preformed metal crowns, and topical fluoride application. The perioperative course was uneventful and no respiratory, airway, or anesthesia-related adverse events were observed. The patient was followed up continuously for 6 to 12 months after dental treatment.

**Conclusion:**

This report delineates the multidisciplinary perioperative management of comprehensive dental treatment in a 4-year-old patient with UCMD, thereby providing a clinical reference for oral healthcare in individuals with this rare disorder.

## 1 Introduction

Ullrich congenital muscular dystrophy (UCMD) is a rare collagen VI-related myopathy most commonly caused by *de novo* autosomal dominant variants, although cases of autosomal recessive inheritance have also been reported (1). Its estimated prevalence is approximately 1.3 per million individuals (2). This condition results from pathogenic variants of COL6A1, COL6A2, and COL6A3, which encode type VI collagen, a vital component of the extracellular matrix in skeletal muscle, skin, and various other tissues. Type VI collagen provides essential structural support to cells and plays a pivotal role in preserving tissue elasticity, vascular integrity, and mechanical stability (1).

UCMD is characterized by progressive and complicated clinical manifestations among affected individuals. The typical features include congenital muscle weakness, hypotonia, proximal joint contractures, and distal joint hyperextensibility, which are frequently accompanied by congenital torticollis, hip dislocation, scoliosis, spinal rigidity and calcaneal prominence. Progressive deterioration diaphragmatic function commonly leads to respiratory insufficiency, often necessitating respiratory support, and predisposing patients to respiratory failure during late childhood or adolescence. Despite the severity of muscular involvement, cognitive and intellectual development typically remain unaffected.

Some studies have shown that patients with muscular dystrophy can not maintain oral hygiene due to progressive decline in muscle function, therefore, suffering from various oral diseases (3–5). Patients with stable muscular dystrophy can safely undergo dental treatment in an outpatient setting. However, those with severe contractures or respiratory compromise should receive treatment under general anesthesia with prudent perioperative monitoring to minimize the risk (3, 6).

A focused literature search was conducted in PubMed, Web of Science, Embase, Scopus, and Google Scholar using combinations of the terms “Ullrich congenital muscular dystrophy,” “UCMD,” “collagen VI-related myopathy,” “dental treatment,” “oral rehabilitation,” “dentistry,” “general anesthesia,” and “general anaesthesia.” Published UCMD-specific reports mainly focus on anesthetic or perioperative management in non-dental procedures, whereas broader muscular dystrophy literature discusses oral health considerations and dental treatment in special healthcare needs populations. To our knowledge, no previous report has described comprehensive dental rehabilitation under general anesthesia in a pediatric patient with UCMD. Here, we present the case of a 4-year-old girl with UCMD who underwent comprehensive dental treatment under general anesthesia.

## 2 Case description

A 4-year-old girl diagnosed with UCMD visited the Department of Stomatology, Chinese Academy of Medical Sciences & Peking Union Medical College Hospital (PUMCH), with a chief complaint of 1-month history of pain that had begun to interfere with eating in the left lower primary molar.

The patient was born at term via cesarean section with a birth weight of 3 kg, and had no history of asphyxia. She was born with bilateral hip dislocation, generalized muscle weakness, and torticollis. She rolled over at 8–9 months and sat independently at 1 year but was unable to crawl or stand unaided. At 18 months of age, genetic testing confirmed the diagnosis of UCMD and revealed a pathogenic COL6A3 variant (c.6210 + 1G > A) and a variant of uncertain significance (c.6413A > G). The patient underwent surgical correction of the right hip while the left hip continued to receive rehabilitation. The parents were non-consanguineous and had no family history of neuromuscular disease.

The patient was 13 kg in weight and 103 cm in height. She was alert with normal language and cognitive development, but was poorly cooperative. Physical examination was as follows: thoracic asymmetry, right-sided scoliosis with a trunk orthosis in place and torticollis due to shortening of the left sternocleidomastoid muscle. She also had reduced muscle bulk, generalized joint hypermobility, and delayed motor development. Limb strength was decreased, graded as 4/5. The feeding was slow and occasionally accompanied by choking episodes. No pathological reflexes were observed. Routine blood tests, renal and liver function tests, and coagulation profiles were within normal limits.

**Figure 1:**
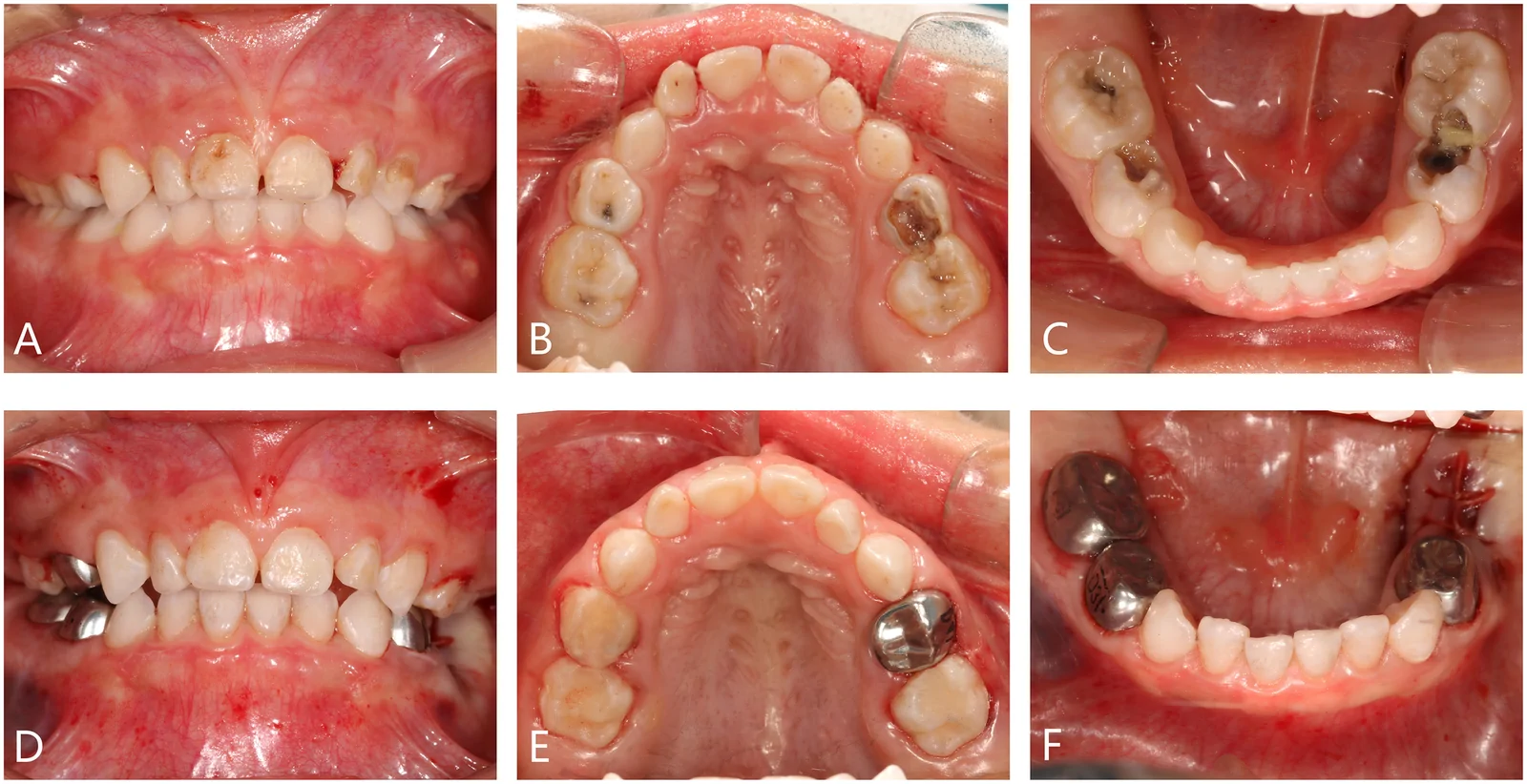
Intraoral photograph before and after dental treatment under general anesthesia. **(A-C)** Preoperative intraoral photographs showing generalized plaque accumulation, marginal gingival inflammation, and multiple carious lesions in the primary dentition. **(A)** Frontal view showing anterior carious involvement and inflamed gingival tissues. **(B)** Maxillary occlusal view showing carious lesions involving the maxillary primary molars and anterior teeth. **(C)** Mandibular occlusal view showing deep carious lesions of the mandibular primary molars, including tooth 75. **(D-F)** Postoperative intraoral photographs after dental treatment. **(D)** Frontal view after restorative treatment. **(E)** Maxillary occlusal view showing resin restorations and a preformed metal crown. **(F)** Mandibular occlusal view showing preformed metal crowns on teeth 74, 84, and 85 and the extraction site of tooth 75.

Dental notation in this report follows the Fédération Dentaire Internationale (FDI) two-digit numbering system. Oral examination revealed generalized gingivitis in primary dentition. Tooth 75 exhibited deep caries with grade II mobility and a gingival sinus tract. Teeth 51, 54, 55, 63, 64, 65, 74, 84, and 85 demonstrated moderate to deep caries without mobility (Figures 1A–C). The patient was uncooperative and could not undergo dental x-ray examination.

### 2.1 Diagnose

The preliminary diagnoses included chronic apical periodontitis of tooth 75, severe early childhood caries, UCMD, torticollis, and scoliosis.

### 2.2 Treatment procedure

**Figure 2:**
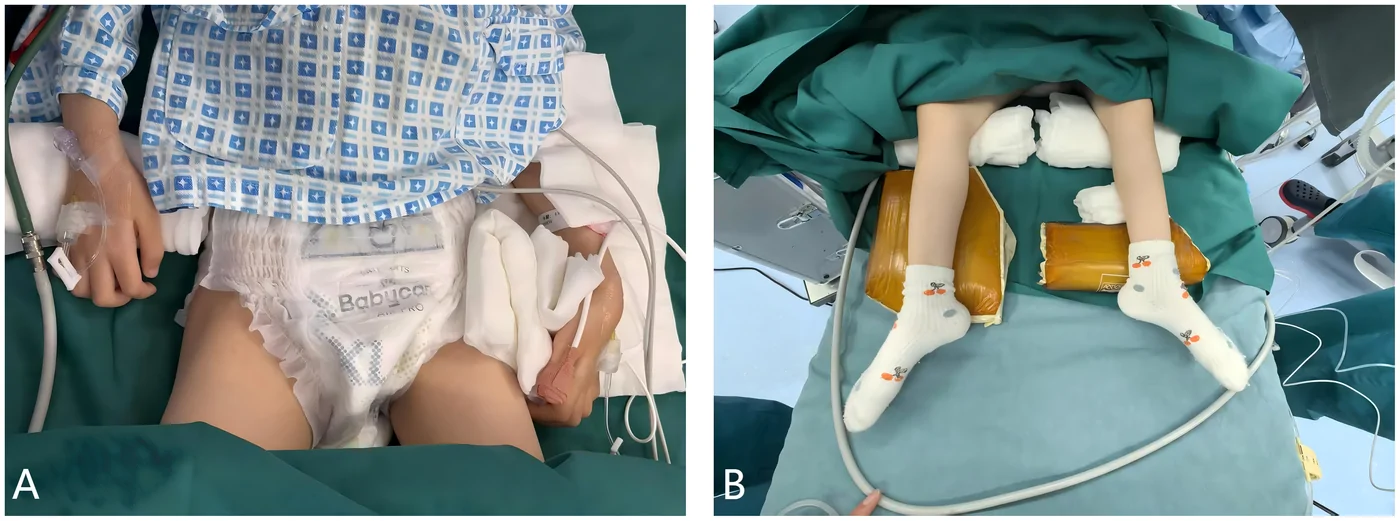
**(A-B)** intraoperative positioning and pressure-point protection during dental treatment under general anesthesia.

Following a multidisciplinary assessment by the pediatric, anesthesiology, and nursing teams, the patient was hospitalized for dental treatment under general anesthesia. The day before operation, the patient was admitted to the pediatric ward. To evaluate the patient’s respiratory function, preoperative arterial blood gas analysis was performed and the result was normal. Her venous access was established after 1-hour of topical anesthesia with 5% compound lidocaine cream. On the day of the operation, anesthesia was induced with propofol, fentanyl and rocuronium and endotracheal intubation was performed via fiberoptic bronchoscopic guidance. Anesthesia was then provided with propofol and remifentani titrated to maintain the bispectral index at 60-80. During the procedure, SpO₂, end-tidal carbon dioxide, respiratory rate, airway pressure, blood pressure, heart rate, body temperature, and bispectral index were continuously monitored. Intraoperative SpO₂ was maintained at 98%–100%, and no hypoxemia, airway obstruction, or hemodynamic instability was observed. Rectal temperature monitoring was performed throughout the operation. Following the administration of general anesthesia, Personalized care was provided for patient to maintain the safe positioning, joint and skin protection (Figure 2).

**Figure 3:**
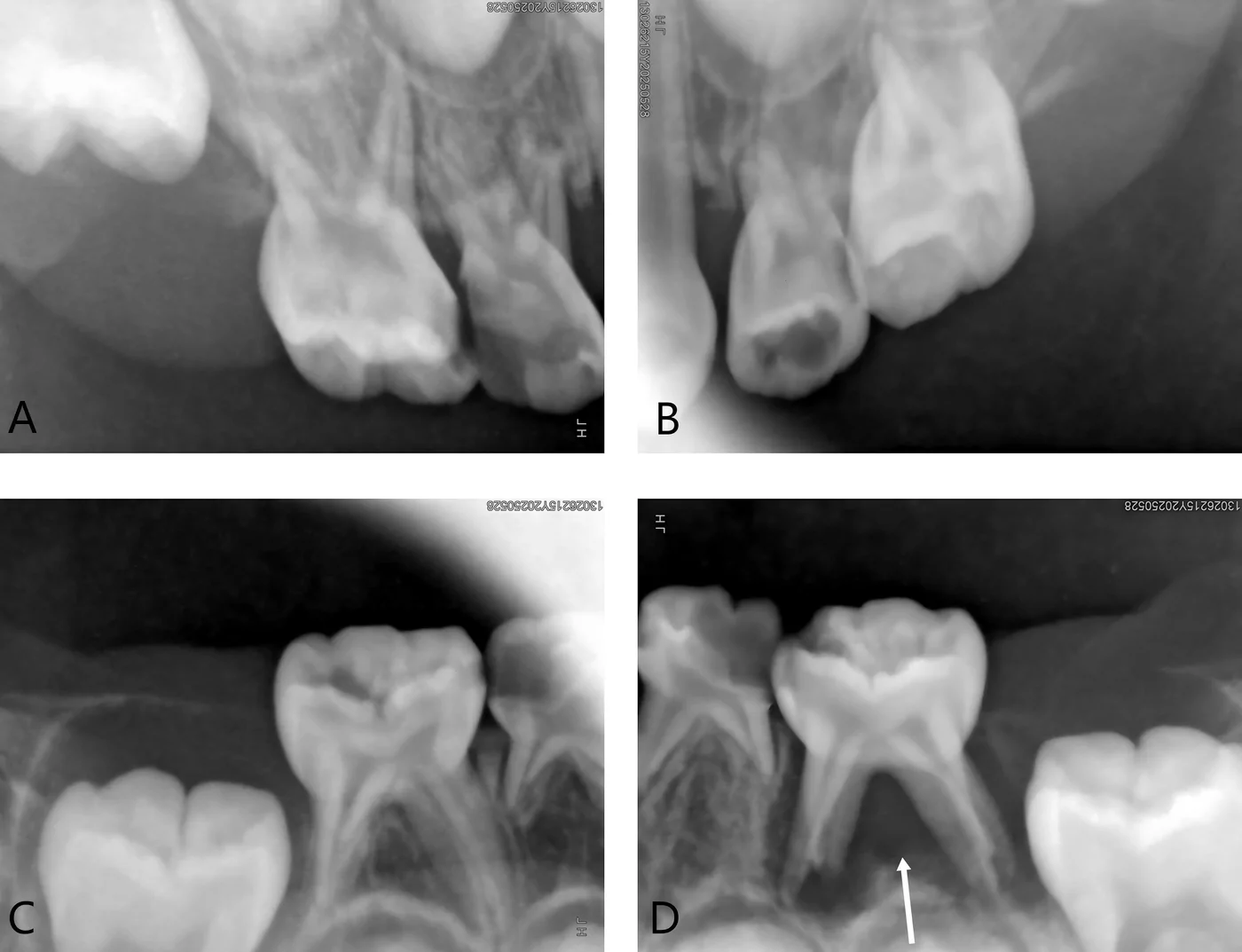
Periapical radiographs obtained after induction of general anesthesia. **(A-C)** Multiple cavitated carious lesions involving the primary molars. **(D)** Tooth 75 showed root resorption and periapical radiolucency. The arrow indicates the radiolucent lesion, with an incomplete corticated border of the developing permanent teeth.

Intraoperative x-ray examnation revealed root resorption and periapical radiolucency of tooth 75 and an incomplete hard bone plate in the permanent teeth (Figure 3). Extraction of tooth 75 followed by suture placement. Pulpotomy followed by preformed metal crowns (PMCs) for teeth 54, 74, 84, and 85; pulpotomy followed by resin restoration for tooth 55; resin restorations for teeth 51, 63, 64, and 65; fluoride varnish was applied on the whole teeth (Figures 1D–F).

The patient successfully completed the 2-hour procedure without any respiratory complications. At the end of the procedure, the patient was extubated after adequate spontaneous breathing and stable vital signs had recovered. Postoperative monitoring included oxygen saturation, respiratory pattern, airway patency, heart rate, blood pressure, consciousness, and swallowing function. Oral intake was resumed 2 hours postoperatively after safe swallowing was confirmed, and no choking was observed. The patient was discharged after 2 days of observation. At discharge, the intraoral wounds had healed well, with no bleeding or infection noted. No postoperative respiratory depression, hypoxemia, airway obstruction, aspiration, or anesthesia-related adverse events were observed.

### 2.3 Six-months follow-up

**Figure 4:**
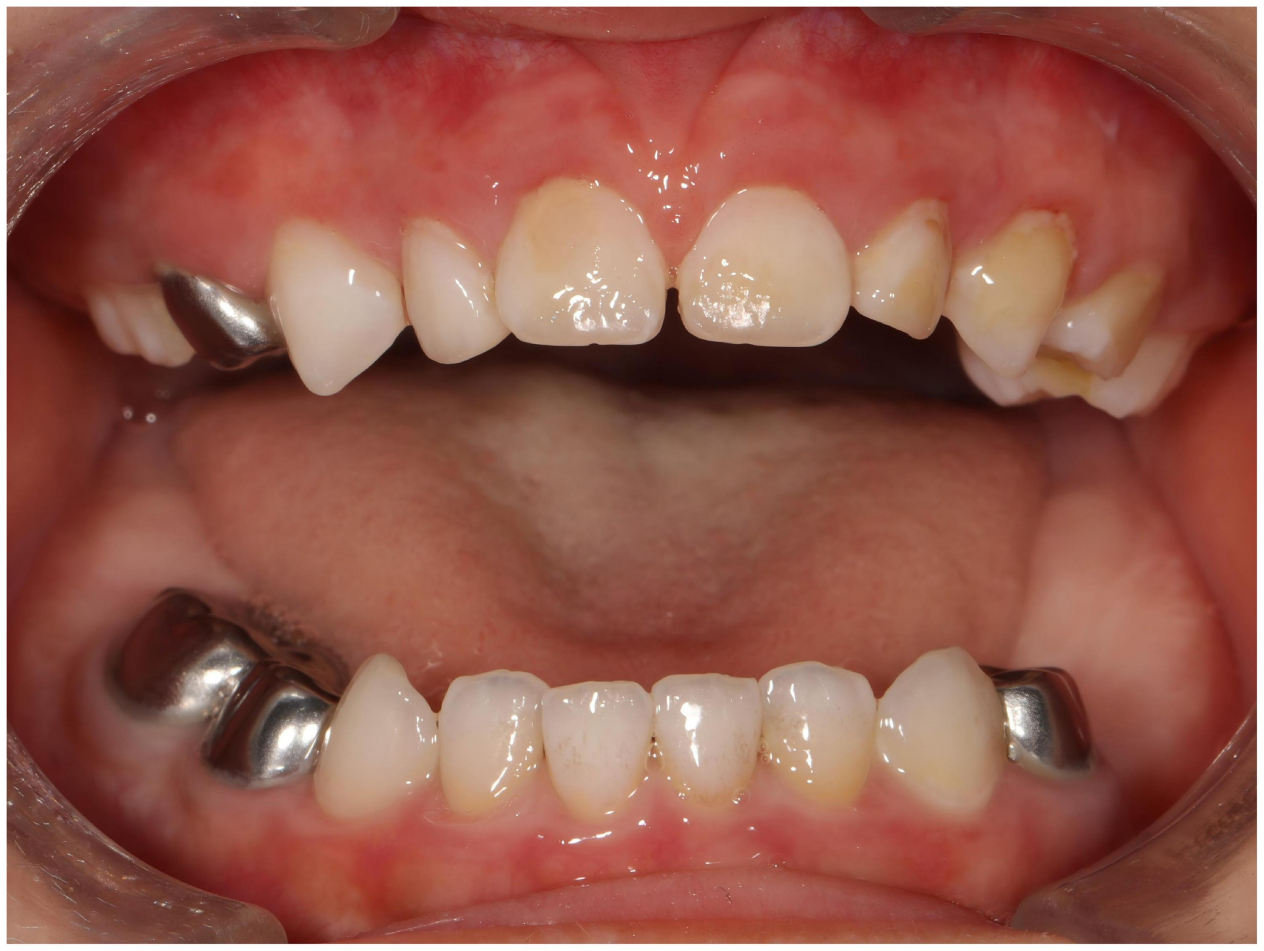
Intraoral photograph at the 6-month follow-up after dental treatment.

Intraoral examination demonstrated poor oral hygiene; healthy gingiva; intact PMCs on teeth 54, 74, 84, and 85; and stable resin restorations in teeth 51, 55, 63, 64, and 65. The extraction site of tooth 75 had healed completely (Figure 4). We gave oral hygiene instruction for parents.

## 3 Discussion

UCMD, first described by Ullrich in 1930, is a rare hereditary myopathy caused by mutations in the genes encoding collagen VI. The condition is heterogeneous and progressive and is characterized by congenital weakness, hypotonia, proximal joint contractures, distal joint hyperextensibility, and frequent skeletal and respiratory complications.

In this case, the patient had multiple teeth to be treated and significant risks such as airway obstruction and aspiration due to prolonged dental treatment in an outpatient setting. Furthermore, the patient cannot cooperate with dental treatment with Frankl Behavior Rating 2 and the preoperative anesthetic risk was classified as American Society of Anesthesiologists class III. Consequently, the patient was admitted to be hospitalized for dental treatment under general anesthesia (3, 6).

One of the major perioperative concerns in such patients is the potential for difficult intubation due to limited mouth opening and cervical spine movement (7–9). Accordingly, we executed fiberoptic bronchoscopic–guided intubation to secure the airway safely and accurately (7). In addition, nasotracheal intubation was chosen to facilitate intraoral instrumentation and dental treatment.

Another key perioperative consideration in patients with UCMD is the selection of anesthetic agents, primarily aimed at mitigating the risk of life-threatening complications such as anesthesia-induced rhabdomyolysis, hyperkalemia, or malignant hyperthermia-like reactions, rather than proven malignant hyperthermia susceptibility, while also accounting for the risk of postoperative respiratory failure (10–15). Current evidence does not establish collagen VI-related myopathy, including UCMD, as a condition with definite malignant hyperthermia susceptibility. Therefore, malignant hyperthermia-like reactions should be distinguished from true malignant hyperthermia. Although inhalational induction to facilitate intravenous access in uncooperative patients with muscular dystrophy has been reported (10, 16), the use of volatile anesthetics in this population remains controversial (13, 17). However, most evidence regarding volatile anesthetic-related rhabdomyolysis or malignant hyperthermia-like reactions is derived from dystrophinopathies rather than UCMD, and data specific to collagen VI-related myopathies remain limited. In particular, the combination of volatile agents and depolarizing neuromuscular blocking drugs (e.g., succinylcholine) has been associated with rhabdomyolysis, hyperkalemia, and malignant hyperthermia-like reactions; therefore, succinylcholine should be strictly avoided in patients with muscular dystrophies (10–13). As a safer alternative, we secured intravenous access preoperatively and administered total intravenous anesthesia with propofol (17–19). For neuromuscular blockade during induction, we selected the non-depolarizing agent rocuronium (20).

To reduce the risk of postoperative respiratory failure attributable to residual anesthetic effects on diaphragmatic and upper airway muscle function, short-acting agents (e.g., remifentanil) are recommended to minimize perioperative opioid exposure, together with vigilant respiratory monitoring (9, 10).

During the dental treatment, tooth 75 was extracted to remove the severe periapical infection of the primary molar and reduce the damage to the successor's permanent tooth. This decision was based on grade II mobility, the presence of a gingival sinus tract, radiographic root resorption, and periapical radiolucency, which indicated poor prognosis for tooth retention. Inflammatory granulation tissue was observed in the furcation area upon extraction. Some studies recommend a distal shoe appliance or removable space maintainers before the eruption of the first permanent molar, however, considering the risk of dislodgement and aspiration of the appliance, uncooperative behavior, and difficulty in maintaining oral hygiene, a distal shoe space maintainer was not placed. In addition, the need for repeated follow-up and appliance adjustment was considered difficult in this patient because of her limited cooperation and special healthcare needs. Therefore, regular observation and early orthodontic intervention after eruption of the mandibular left first permanent molar were considered safer and more practical. Early orthodontic intervention will be planned after the eruption of the mandibular left first permanent molar (21). In the teeth with extensive lesions but without clinical mobility or radiographic evidence of advanced periapical infection, pulpotomy followed by PMCs was selected for teeth 54, 74, 84, and 85 after communication with the pediatrician and guardian confirmed the absence of planned magnetic resonance imaging of the head region in the near future (22). Tooth 55 was treated with pulpotomy followed by resin restoration because sufficient remaining tooth structure allowed direct restoration after pulp therapy. Teeth 51, 63, 64, and 65 were restored with resin because the carious lesions were less extensive and did not require full coronal coverage. PMCs can offer excellent coronal coverage and are better over time than multisurface intracoronal restorations,therefore, PMCs are strongly recommended for children at high risk of dental caries undergoing dental treatment under general anesthesia (22).

Considering the risk of aspiration and delayed recovery of swallowing after anesthesia, oral intake was cautiously initiated with small sips of water after 2 h, followed by a semi-liquid diet once safe swallowing was confirmed. Children with UCMD may remain at high risk of oral disease because progressive motor impairment can limit effective toothbrushing, dietary control, and regular dental attendance. In this patient, long-term concerns include recurrent caries, gingival inflammation, restoration failure, and plaque accumulation around preformed metal crowns and erupting permanent teeth. Therefore, preventive care should be caregiver-centered and risk-based. Caregiver-assisted toothbrushing with fluoridated toothpaste, dietary counseling, professional fluoride application every 3–6 months, regular review of restorations and crowns, and monitoring of permanent tooth eruption are recommended (23). The recall interval should be individualized according to caries risk, oral hygiene, cooperation, swallowing function, and respiratory status (23, 24).

## 4 Conclusions

To our knowledge, this is the first reported case of comprehensive dental treatment under general anesthesia in a pediatric patient with UCMD. This case underscores the potential anesthetic and perioperative risks in this patient population, emphasizing the significance of multidisciplinary collaboration among the dental, anesthesiology, pediatric, and nursing teams. Thorough knowledge of disease pathology and tailored planning can promote safer and more effective oral care, thereby improving masticatory function and the overall quality of life in children with rare neuromuscular disorders.

## Data Availability

The original contributions presented in the study are included in the article/Supplementary Material, further inquiries can be directed to the corresponding authors.
